# Clinical Researches on Auscultation of the Respiratory Organs, and on the First Stage of Phthisis Pulmonalis

**Published:** 1842-01

**Authors:** 


ART. VIII.—*Clinical Researches on Auscultation of the Respiratory Organs, and on the First Stage of Phthisis Pulmonalis.* By JULES FOURNET. Translated from the French by THOMAS BRADY, M.B. Fellow and Professor of Medical Jurisprudence in the King and Queen's College of Physicians in Ireland. Part I.—*London,* 1841. 8vo, pp. 227.

WE gave so extensive a review of M. Fournet's original work in a former number of this Journal (vol. IX. p. 299), to which we refer the reader, that nothing remains for us to say except that the translation is an excellent one, and that the notes supplied by Dr. Brady add considerably to the value of the treatise. They whom our analysis does not content, and who are not in possession of the original volume, ought assuredly to place the English version on their shelves.

